# Extracellular Vesicles as Signal Carriers in Malignant Thyroid Tumors?

**DOI:** 10.3390/ijms23063262

**Published:** 2022-03-17

**Authors:** Małgorzata Grzanka, Anna Stachurska-Skrodzka, Anna Adamiok-Ostrowska, Ewa Gajda, Barbara Czarnocka

**Affiliations:** 1 Department of Biochemistry and Molecular Biology, Centre of Postgraduate Medical Education, Marymoncka 99/103, 01-813 Warsaw, Poland; anna.adamiok@cmkp.edu.pl (A.A.-O.); ewa.gajda@cmkp.edu.pl (E.G.); 2 Department of Cell Biology and Immunology, Centre of Postgraduate Medical Education, Marymoncka 99/103, 01-813 Warsaw, Poland; anna.stachurska@cmkp.edu.pl

**Keywords:** thyroid cancer, extracellular vesicles, tetraspanins, angiogenesis

## Abstract

Extracellular vesicles (EVs) are small, membranous structures involved in intercellular communication. Here, we analyzed the effects of thyroid cancer-derived EVs on the properties of normal thyroid cells and cells contributing to the tumor microenvironment. EVs isolated from thyroid cancer cell lines (CGTH, FTC-133, 8505c, TPC-1 and BcPAP) were used for treatment of normal thyroid cells (NTHY), as well as monocytes and endothelial cells (HUVEC). EVs’ size/number were analyzed by flow cytometry and confocal microscopy. Gene expression, protein level and localization were investigated by qRT-PCR, WB and ICC/IF, respectively. Proliferation, migration and tube formation were analyzed. When compared with NTHY, CGTH and BcPAP secreted significantly more EVs. Treatment of NTHY with cancer-derived EVs changed the expression of tetraspanin genes, but did not affect proliferation and migration. Cancer-derived EVs suppressed tube formation by endothelial cells and did not affect the phagocytic index of monocytes. The number of 6 μm size fraction of cancer-derived EVs correlated negatively with the CD63 and CD81 expression in NTHY cells, as well as positively with angiogenesis in vitro. Thyroid cancer-derived EVs can affect the expression of tetraspanins in normal thyroid cells. It is possible that 6 μm EVs contribute to the regulation of NTHY gene expression and angiogenesis.

## 1. Introduction

Thyroid cancer accounts for approximately 1% of all malignancies [1]. However, it is the most common endocrine neoplasm and its incidence has been increasing in recent years, especially in women [2]. According to histopathological criteria, thyroid carcinomas are classified as papillary thyroid carcinoma (PTC); follicular thyroid carcinoma (FTC); anaplastic thyroid carcinoma (ATP); medullary thyroid carcinoma (MTC); poorly differentiated thyroid carcinoma (PDTC); or Hurtle cell carcinoma [3]. FTC and PTC variants account for up to 90% of all thyroid tumor cases [4,5,6]. 

Extracellular vesicles (EVs) are small membranous structures, secreted by both normal and tumor cells [7,8,9]. The size varies greatly, from the smallest exosomes (30–300 nm) through large oncosomes (1–10 µm) to giant vesicles (3–42 µm). The size, role and cargo of EVs depend on the type of cells from which they are released [10,11]. The highest EVs’ concentrations are secreted by cancer cells [10,12,13]. Enhanced EV release has also been shown in autoimmune diseases [14].

Extracellular vesicles are formed by budding out of the cell through the cell membrane [15,16]. Therefore, the membrane that surrounds these vesicles has a very similar composition to that of the cell membrane [7,10]. However, it can have up to 100 times higher concentrations of the membrane proteins than are found in cell lysates [16]. EV release is induced by various factors, including hypoxia [17,18] and other stress conditions [19].

EVs contain lipids and nucleic acids, including mRNA and miRNA [15,16,17,18,20,21], as well as many protein markers, such as Alix [22,23,24], Caveolin-1 [25] and tetraspanins [8,26,27,28]. Caveolin-1 has been shown to be overexpressed in many cancer types [25], while tetraspanins are involved in essential biological processes such as cell adhesion, proliferation, apoptosis and angiogenesis [29,30,31,32,33,34]. They can also modulate the immune response [10,35,36] as well as affect tumor progression by enhancing invasiveness [32,33,37,38] and metastatic potential [31,39]. Moreover, EVs contain proteins of the ezrin-radixin-moesin (ERM) family which mediate the interaction of tetraspanins with the cytoskeleton and influence cell migration and metastasis, contributing to tumorigenesis and cancer progression [40,41,42].

EVs play a key role in intercellular communication [12,15] and can also stimulate or suppress the immune system [10]. The exchange of information between cells can take place in three ways: (1) direct stimulation of target cells, by ligands provided by EVs; (2) transfer of surface receptors from one cell to another; (3) epigenetic reprogramming of the target cell by delivery of proteins, mRNAs and/or transcription factors [10,43]. Thus, EVs can regulate recipient cells at the post-transcriptional level [17]. EVs secreted by cancer cells are of particular interest as enhancers of angiogenesis and migration [7,11,18,44], or activators of anti-tumor T lymphocyte apoptosis [44]. In addition, tumor-derived EVs have the ability to transform normal cells in the tumor microenvironment to promote tumor progression [13] and confer resistance to chemotherapeutics [17,45]. Some EVs derived from metastatic cells can build a niche for further metastases [8]. 

The biological role of EVs appears to be different in different types of cancer. It was suggested that exosomal small RNAs (miRNAs, circRNAs) and proteins isolated from plasma or serum can serve as useful diagnostic and/or prognostic biomarkers for patients with thyroid nodules [46,47] and papillary thyroid carcinoma [48,49,50,51,52]. Furthermore, it was shown that the exosomal small and long RNAs and proteins can affect the progression of PTC in vitro and/or in vivo [49,52,53,54,55,56,57]. However, to our knowledge no detailed studies have been conducted on the role of EVs in the course of follicular, papillary and anaplastic thyroid cancers as well as their effect on the surrounding normal thyroid follicular cells. Therefore, in this study we aimed to quantify the extracellular vesicles released by cells of different types of thyroid cancer, determine the expression level of genes involved in EV formation and to determine the effect of EVs released by thyroid cancer cells on the morphology, viability and invasive potential of healthy thyroid cells, as well as on monocytes and endothelial cells representing the tumor microenvironment. 

## 2. Results

### 2.1. Isolation and Characterization of EVs

**Figure 1:**
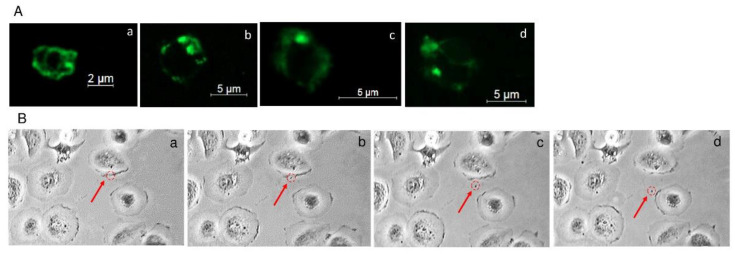
(**A**) EVs from different cell lines labeled with Caveolin-1 and imaged by confocal microscopy: (**a**,**b**)—EVs isolated from CGTH; (**c**)—EVs isolated from TPC-1; (**d**)—EVs isolated from FTC-133, (green—Alexa Fluor 488); (**B**) Representative images of EVs’ release from the 8505c cell line (automatic sequential microscopic image registration (**a**–**d**) arrow—released EV).

All the cell lines included in the study (Supplementary Table S1) released extracellular vesicles which were ring-shaped membranous structures between 1 μm and 6 μm in diameter. The resolution of the microscope and the cytometer did not allow for a clear visualization of the smaller EVs or the calculation of their number. No differences were observed between the parameters of the EVs and the cell line from which they were released (results not shown). Selected EVs visualized by confocal microscopy are shown in Figure 1A. The release process of EVs from the cells was observed under a light microscope and recorded using an automatic sequential microscopic image registration system (Figure 1B) No significant differences in the rate and number of released microvesicles were observed between the different cell lines. 

**Figure 2:**
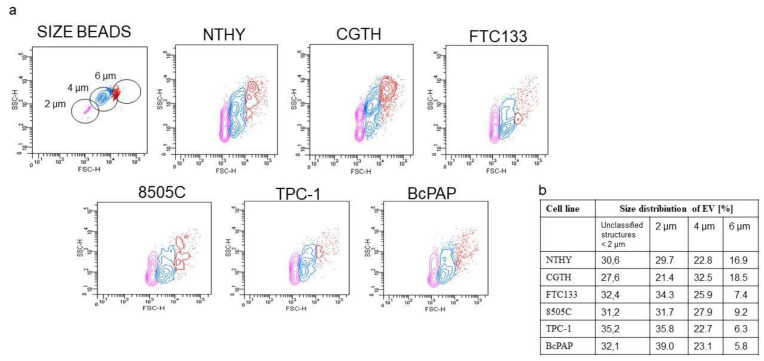
Analyzing the sizes of extracellular vesicles (EVs) released by thyroid cancer cell lines. (**a**). Gating strategy for analyzing EV sizes. Visualization of size beads (with diameters of 2, 4, and 6 μm) shown in a forward scatter (FSC) and side scatter (SSC); (**b**). Size distribution of EVs released by the different thyroid cell lines. NTHY: cell line derived from normal human thyroid follicular cells; CGTG: Thyroid gland squamous cell carcinoma; FTC-133: follicular thyroid carcinoma; 8505c: anaplastic thyroid carcinoma; TPC-1: thyroid gland papillary carcinoma; BcPAP: thyroid gland papillary carcinoma. Full characteristics of the used cell lines are provided in Supplementary Table S1.

The analysis of extracellular vesicles using calibration beads showed that the EV diameters ranged from 2 to 6 µm (Figure 2a). Each of the cell lines tested released EVs of varying sizes. The BcPAP cells released the highest fraction of EVs with a diameter of 2 μm, while the CGTH cells released the smallest fraction of these EVs. In contrast, CGTH cells secreted mostly EVs with a diameter of 6 μm, whereas BcPAP released the smallest percentage of these EVs. The number of secreted EVs of intermediate size (4 μm) was comparable in all tested lines (Figure 2b).

**Figure 3:**
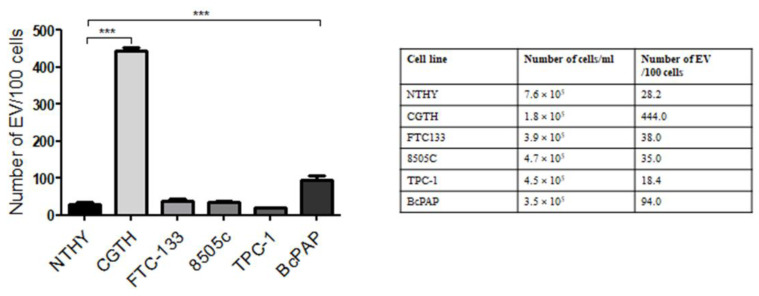
The average number of extracellular vesicles (EVs) released by thyroid cell lines per 100 cells. Data are shown as means with standard deviations (±SEM). ***: *p* < 0.001.

The cytometric analysis also showed that the numbers of secreted EVs differed between cell lines. The number of EVs per 100 cells ranged from 18.4 (TPC-1 cell line) to 444 (CGTH cell line) (Figure 3).

### 2.2. Thyroid-Derived Cells Express EV Markers

**Figure 4:**
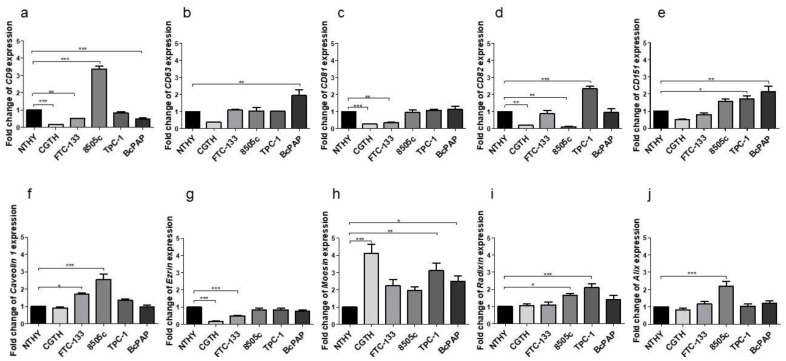
Relative expression of tetraspanins (**a**–**e**); Caveolin-1 (**f**); Ezrin (**g**); Moesin (**h**); Radixin (**i**); and Alix (**j**) in thyroid cell lines. Data are reported as means with standard deviation (±SEM). *: *p* < 0.05; ** *p* < 0.01; ***: *p* < 0.001.

The expression of the canonical EV markers such as tetraspanins, Caveolin-1, Alix and ERM proteins was confirmed using qRT-PCR and confocal microscopy in all the analyzed thyroid cell lines. (Figure 4 and Supplementary Figures S1 and S2).

Next, the expression levels of the canonical EVs markers: tetraspanin; caveolin-1; Alix and ERM proteins, were analyzed (Figure 4).

The expression levels were diverse between the cell lines. The expression level of the CD9 gene was significantly lower in CGTH (*p* < 0.001), FTC-133 (*p* < 0.01) and BcPAP (*p* < 0.05) cell lines, and higher in the 8505c cells compared to the normal thyroid cell line (NTHY; *p* < 0.01) (Figure 4a). The CD63 gene expression was significantly decreased in the CGTH line compared to the NTHY cells (*p* < 0.05). (Figure 4b). The CD81 expression was statistically significantly lower in the CGTH and FTC-133 cell lines compared to NTHY cells (*p* < 0.01 for both comparisons) (Figure 4c). In contrast, the tetraspanin CD82 expression levels were lower in the CGTH and 8505c cells (*p* < 0.01), and significantly higher in TPC-1 cells (*p* < 0.05) compared to NTHY cells (Figure 4d). The expression of tetraspanin CD151 was not significantly different between the cell lines (Figure 4e). The expression of caveolin-1 was statistically significantly higher in FTC-133 (*p* < 0.05) and 8505c (*p* < 0.001) cell lines compared to NTHY cells (Figure 4f). The expression levels of Alix (Figure 4j), another EV marker, was similar between the NTHY cells and thyroid cancer cell lines, except for 8505c, in which the Alix expression was higher than in the NTHY cells. Ezrin expression (Figure 4g) was statistically significantly lower in the CGTH (*p* < 0.01) and FTC-133 (*p* < 0.05) cells than in NTHY cells, whereas Moesin expression (Figure 4h) was significantly higher in all tumor cell lines tested (*p* < 0.05 for FTC-133 and *p* < 0.01 for CGTH, 8505c, TPC-1 BcPAP). The expression of radixin (Figure 4i) was statistically significantly higher in the 8505c, TPC-1 and BcPAP cells compared to the NTHY cell line (*p* < 0.05).

### 2.3. The Effect of Thyroid Cancer-Derived EVs on the Functioning of Normal Thyroid Cells

**Figure 5:**
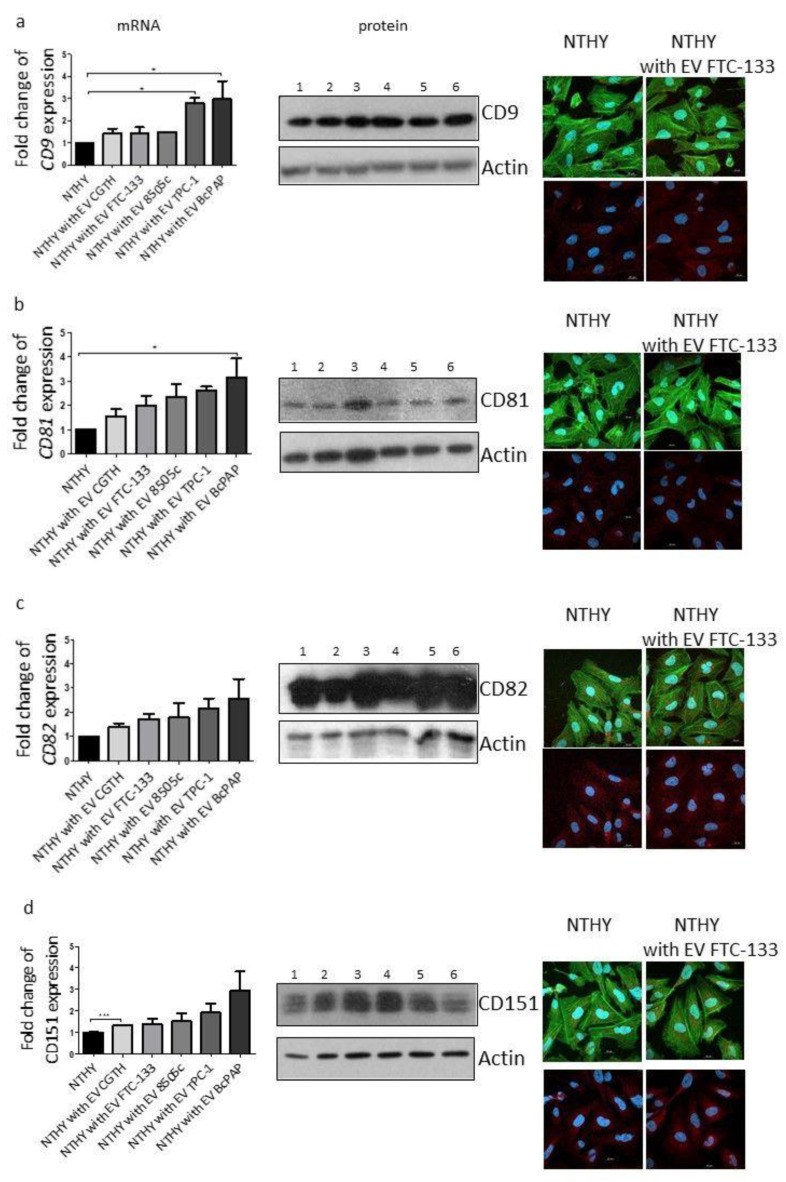
The effect of EVs derived from thyroid cancer cells on the expression of tetraspanins in NTHY cells. The expression of tetraspanins is shown in separate panels: CD9 (**a**); CD81 (**b**); CD82 (**c**); and CD151 (**d**). Each panel shows: the level of gene expression (qRT-PCR) (the graphs placed on the left side of the figure); the level of protein expression (WB) (the images placed in the middle); the representative images of ICC/IF analysis (the microscopic photographs shown on the right side of the figure). Line 1: NTHY alone; 2: CGTH; 3: FTC-133; 4: 8505c; 5: TPC-1, 6: BcPAP. Data are shown as means with standard deviation (±SEM). *: *p* < 0.05, ***: *p* < 0.001.

The effects of EVs derived from thyroid cancer cells on the function of normal thyroid cells, NTHY, were then analyzed. To this effect, the expression of the canonical EV markers by these cells was analyzed following a five-day incubation with cancer-derived EVs. The expression levels of CD9 were significantly higher in the NTHY cells cultured with EVs isolated from TPC-1 (*p* < 0.05) and BcPAP (*p* < 0.05) cells, compared with the control NTHY cells (not incubated with EVs; Figure 5a). No difference in the CD63 expression was observed (data not shown). NTHY cells that were incubated with EVs isolated from BcPAP showed a statistically significantly higher expression of CD81 (*p* < 0.05) compared to the control NTHY cells (Figure 5b). The expression levels of CD82 did not differ from those in the NTHY cells cultured with EVs (Figure 5c). CD151 expression was significantly higher in cells cultured with EVs isolated from CGTH cells (*p* < 0.001) compared to untreated cells (Figure 5d). The protein expression of all tetraspanins is shown in Figure 5c and in Supplementary Figure S3.

**Figure 6:**
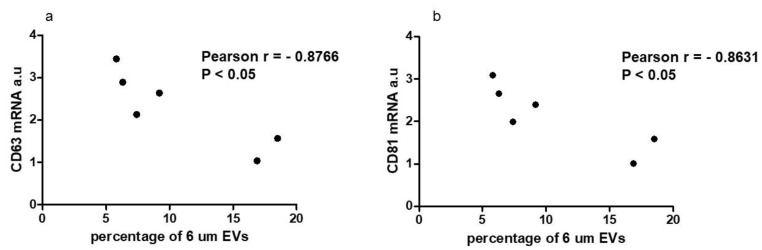
The number of cancer-derived 6 μm EVs correlates with the expression of tetraspanins. The plots show the Pearson correlation between the expression levels of CD63 (**a**); and CD81 (**b**) in NTHY cells (after incubation with cancer-derived EVs) and the percentage of 6-µm extracellular vesicles (EVs) released by thyroid cancer cells.

Incubating NTHY cells with EVs isolated from thyroid cancer cell lines did not affect the Alix, Moesin and Radixin expression (data not shown). Caveolin-1 expression was significantly higher in the NTHY cells incubated with EVs isolated from BcPAP cells (*p* > 0.05), while Ezrin expression levels were higher in the NTHY lines incubated with EVs secreted by 8505c cells (data not shown). Next, we searched for the potential functional associations between the specific EVs’ fractions and the observed effect in changes of tetraspanin expression. To this end we analyzed the correlations between the numbers of EVs of specific sizes released by thyroid cancer cells and the expression of tetraspanins in the NTHY cells treated by the EVs. We investigated the correlation between the levels of EVs released by the thyroid cancer cell line and the expression of selected genes in the NTHY cells which were incubated with EVs. The analyses showed that there was a negative correlation between the percentage of 6 µm EVs and the expression levels of CD63 and CD81 (Figure 6).

**Figure 7:**
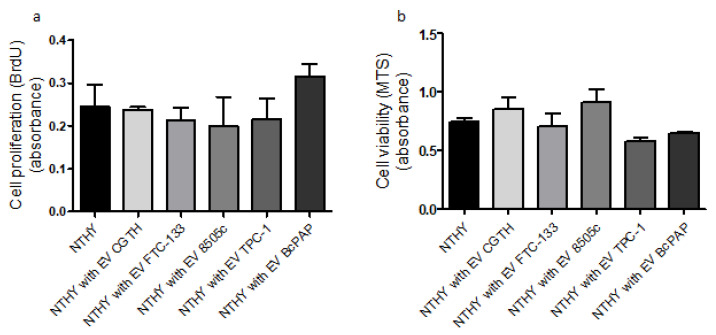
The effect of thyroid cancer-derived extracellular vesicles (EVs) on NTHY (normal thyroid) cell proliferation and viability. (**a**): cell proliferation results by BrdU assay; (**b**): cell viability results by MTS assay.

Compared with the control NTHY cells, the proliferation was enhanced in the NTHY cells cultured with BcPAP-derived EVs, but the differences were not statistically significant. Furthermore, no effect of TPC-1 EVs on NTHY cell proliferation was observed. There were also no statistically significant differences in the viability of cells incubated with EVs (Figure 7).

No statistically significant differences in migration (Supplementary Figure S4) and invasion (Supplementary Figure S5) of NTHY cells and NTHY cells incubated with EVs released from thyroid cancer cell lines were observed, either. In the same line, the wound test showed that incubation with EVs did not statistically significantly change the migration capacities of NTHY cells (Supplementary Figure S6).

### 2.4. Thyroid Cancer EVs Affect Cells of Tumor Microenvironment

**Figure 8:**
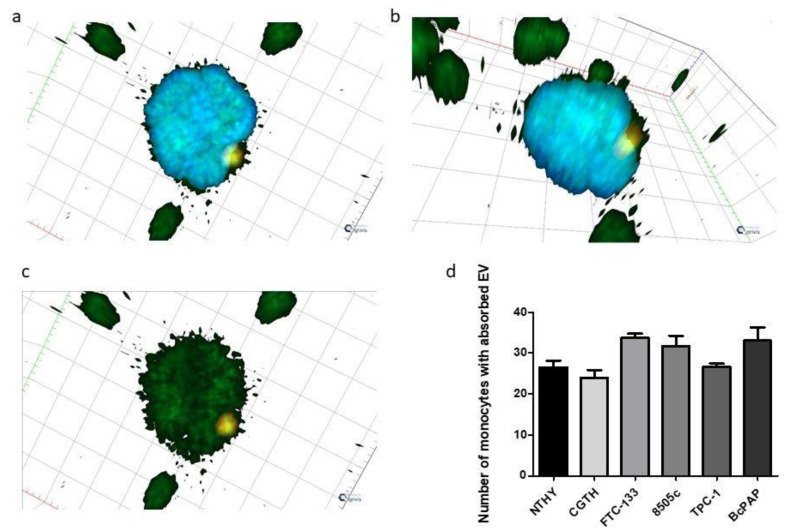
Internalization of thyroid cancer-derived extracellular vesicles (EVs) by monocytes. (**a**–**c**): a 3D model of internalized EVs visualized by confocal microscopy (Zen software—ZEISS). Actin has been stained with Phalloidin-FITC (green), EV Caveoiln-1 with AF 594 (red), and nucleic DNA with DAPI (blue). (**a**,**b**): a monocyte with internalized EV in two different positions; (**c**): a monocyte with internalized EV without a channel for nucleus. (**d**): numbers of monocytes with absorbed EVs, released by different thyroid cell lines.

Finally, we investigated the interactions between human monocytes and extracellular vesicles released from thyroid tumor cell lines. Our results showed that EVs were internalized by monocytes. The XZ and YZ images demonstrated that extracellular vesicles were internalized into the cytoplasm of monocytes (Figure 8a,b), but they were not subsequently internalized by the monocyte nuclei (Figure 8c). Furthermore, we observed that there was no statistically significant differences in the phagocytic index between cell lines (Figure 8d).

**Figure 9:**
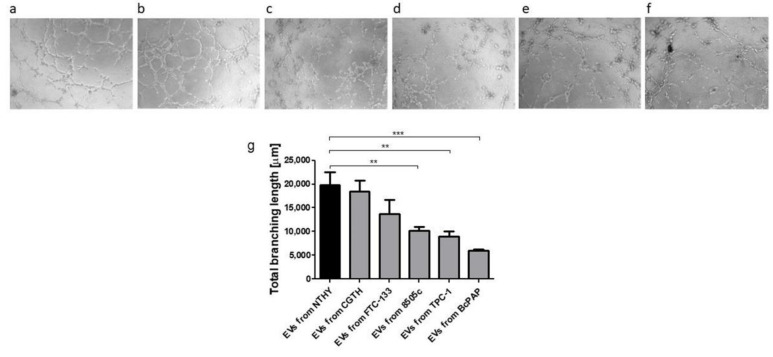
The effect of thyroid-derived EVs on angiogenesis. Upper panel: microscopic analysis of tube formation assay after 5 h: HUVEC cells incubated with: (**a**)—EVs from NTHY; (**b**)—EVs from CGTH; (**c**)—EVs from FTC-133; (**d**)—EVs from 8505c; (**e**)—EVs from TPC-1; (**f**)—EVs from BcPAP; Lower panel: (**g**)—total branching length (Data are reported as mean ± SEM, **: *p* < 0.01, ***: *p* < 0.001).

To investigate the proangiogenic effect of EVs released from NTYH and thyroid cancer cell lines, a human endothelial cell tube formation assay (HUVECs) was performed using the mentioned EVs (Figure 9). HUVECs were incubated on Matrigel^®^-coated plates with EVs derived from NTHY cell lines and tested thyroid cancer lines. In the experimental setup used, HUVECs showed the ability to form tubules by EVs released from NTHY and from EVs of the thyroid cancer lines. In comparison with the level of tubule formation from EVs derived from NTHY, the level of tubule formation from EVs released from thyroid cancer cells was progressively lower after the EVs released from CGTH, FTC-133, 8505c, TPC-1 and BcPAP cell lines. 

**Figure 10:**
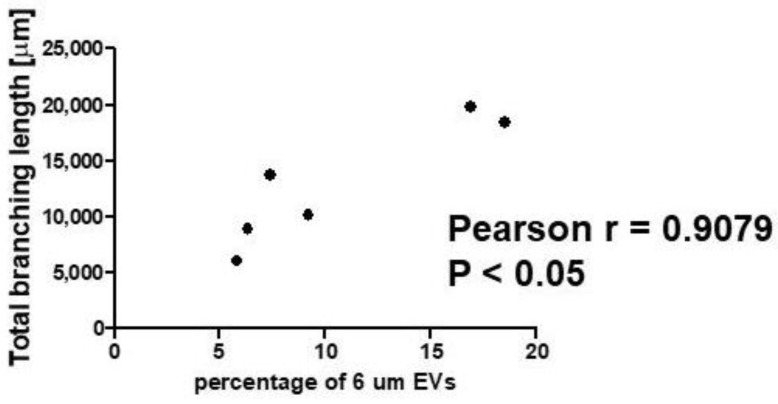
Pearson correlation between the percentage of 6-µm extracellular vesicles (EVs) released by cells and their pro-angiogenic potential.

In contrast, no correlation was observed for other EVs’ fractions (Supplementary Figure S7). The analysis of a correlation between EV numbers and the total length of branches in the tube formation assay showed a positive correlation between the percentage of 6-µm EV and the pro-angiogenic potential of the cells (Figure 10). In contrast, no correlation was observed for the other EV fractions (Supplementary Figure S7).

## 3. Discussion

Extracellular vesicles (EVs) secreted by tumor cells carry proteins, lipids and nucleic acids [15,16,18,21,26] They can modify the genotype and phenotype of normal target cells [10], also at the post-transcriptional level [26,43]. EVs released by cancer cells can affect normal cells to stimulate tumor progression [13]. The role of EVs in thyroid cancer is poorly understood. Several studies indicated that exosomal RNAs and proteins can contribute to the progression of thyroid cancer by acting in an autocrine manner, i.e., by influencing thyroid cancer cells [49,52,53,54,55,56,57]. However, to the best of our knowledge, the effect of thyroid cancer EVs on the functioning of normal thyroid cells remains largely unknown. To date, only two studies on this topic have been published: one reported that exosomes derived from papillary thyroid cancer cells inhibited the proliferation of NTHY cells [55], and the other that exosomes released by thyroid cancer stem cells induced the proliferation and invasive abilities of NTHY cells [57]. 

Here, we found that EVs released by thyroid cancer cells of different types did not stimulate proliferation, migration, invasion, or viability of normal thyroid follicular cells. The key difference between previously published data [55,57] and our study is that we focused on the whole pool of EVs, without separating them into fractions. This way, we were able to test the net effect of multiple EV types on the functioning of normal thyroid cells, which reflects the physiological situation. The calibration beads used for the cytometric measurements allowed us to characterize only the vesicles that had a size of 2, 4 and 6 µm. This means that the EVs pools that we used for treatment of normal thyroid cells included not only the vesicles of 2, 4 and 6 µm, but also—presumably—the smaller vesicles. We found that the EVs derived from thyroid cancer cells affected the expression of tetraspanins in normal thyroid cells, indicating that the latter responded to the presence of EVs in cell culture media. Tetraspanins are multifunctional transmembrane proteins involved, among others, in the regulation of adhesion, integrin signaling, metalloproteinase activity and antigen presentation. They can also act as tumor suppressors or oncogenic proteins by regulating proliferation, migration, invasion and epithelial-mesenchymal transition (EMT) [58,59,60,61,62]. It is worth noting, however, that many tetraspanins have different functions depending on the type of cancer. For instance, CD9 tetraspanin may act as a suppressor in some tumors and as a promoter of oncogenesis in others [63]. The knowledge of the role of tetraspanins in thyroid cancer is limited. Previous reports showed that the expression of the CD9 tetraspanin correlated with lymph node metastases in papillary thyroid microcarcinoma [37]. Remarkably, it was shown that CD82 (known also as KAI1) showed increased expression in papillary thyroid carcinoma but decreased in anaplastic carcinoma [64], which was also confirmed in our study (Figure 4d shows the suppression of CD82 in the 8505c cell line which is derived from anaplastic thyroid carcinoma). The reduction in CD82 levels may be related to the increased metastatic potential [28] which is characteristic of anaplastic cancers. On the other hand, it was suggested that tetraspanin CD63 does not play a direct role in thyroid cancer [28]. However, the presence of the BRAF V600E mutation (characteristic of the BcPAP cell line) is associated with a higher CD63 expression level [65], which was also observed in our study. In our study, the incubation of NTHY cells with EVs isolated from two thyroid cancer cell lines, TPC-1 and BcPAP, stimulated the expression of CD9, suggesting a possible protumorigenic effect. The observed EVs-induced changes in expression of tetraspanins suggested that thyroid cancer-derived vesicles could possibly affect the functioning of normal thyroid cells. However, we did not observe statistically significant differences in proliferation, migration, invasion or viability of NTHY cells treated with cancer-derived EVs. Since the effect of EVs depends on their dose [66], this may possibly result from too short an incubation time which was sufficient to observe changes in tetraspanin mRNA expression, but not sufficient to see the effects on the functioning of NTHY cells. Tetraspanins are also involved in EV biogenesis, uptake and cargo selection [40]. This may possibly suggest that thyroid cancer-derived EVs may affect formation and release of EVs by normal thyroid cells. The possible effects of thyroid cancer-derived EVs on the functioning of normal thyroid cells require further exploration. 

We observed that the whole pool of EVs isolated from thyroid cancer cells sup-pressed angiogenic potential of endothelial cells. However, the positive correlation between the percentage of 6-μm EVs released by different thyroid cancer cells and the total branching length observed in endothelial cells treated with cancer-derived EVs suggested that the 6 μm EVs could promote angiogenesis, in contrast to the EVs of other sizes confined in the whole pool of EVs secreted by thyroid cancer cells. This in turn may possibly suggest that the 6-μm EVs could specifically exert a pro-angiogenic effect, which is not visible when taking into account the entire EV pool, all sizes included. This result is similar to the results by Wu F. et al. who showed that exosomes isolated from papillary thyroid cancer stimulated angiogenesis [67]. Interestingly, we found that the share of the same 6-μm EV fraction was negatively correlated with the expression of CD63 and CD81 tetraspanins in NTHY cells exposed to EVs. This may suggest that the cargo of 6-μm EVs may contain specific regulatory proteins and/or non-coding RNAs specifically affecting angiogenesis and tetraspanin expression. This hypothesis requires further experimental evaluation.

## 4. Materials and Methods

### 4.1. Cell Lines

Six human thyroid cell lines were used in the study. Nthy-ori 3-1 (thyroid follicular epithelial cells, further referred to as NTHY) and follicular thyroid carcinoma cells (FTC-133) were obtained from the European Collection of Authenticated Cell Cultures, ECACC, UK CGTH-W-1 (thyroid gland squamous cell carcinoma, a cell line derived from SW-579 further referred to as CGTH), and BcPAP (derived from papillary carcinoma) were obtained from the German Collection of Microorganisms and Cell Cultures, DSMZ, Braunschweig, Germany). The 8505C (derived from anaplastic carcinoma) and TPC-1 (derived from papillary carcinoma) were generously provided by Dr C. Hoang-Vu (Martin Luther University, Halle, Germany) and by Dr M. Santoro (University of Naples Federico II, Italy), respectively. Full characteristics of the used cell lines are provided in Supplementary Table S1.

NTHY, CGTH and TPC-1 cells were cultured in complete RPMI-1640 medium (Thermo Fisher Scientific, Rockford, IL, USA), FTC-133 and 8505c cell lines in DMEM/F12 medium (ThermoFisher Scientific, Rockford, IL, USA), and BcPAP cell line in complete DMEM/GlutaMAX^™^ (Thermo Fisher Scientific), all supplemented with 10% heat-inactivated fetal bovine serum (FBS, ThermoFisher Scientific, Rockford, IL, USA), at 37 °C in a humidified 5% CO_2_ atmosphere. The number of viable cells was estimated by Trypane Blue exclusion test with automatic counting on an EVA Automatic Cell counter (Nano EnTek, Seoul, Korea). All cell lines used in this study were regularly tested for the presence of mycoplasma contamination. 

### 4.2. Extracellular Vesicles Isolation

Cell cultures were trypsinized, resuspended in medium containing FBS exosome-depleted (Thermo Fisher Scientific, Rockford, IL, USA), seeded on T-25 flasks at a density of 3.7 × 10^5^–5 × 10^5^ cells. After 24 h, when the cells reached 70% confluence, the cultures were centrifuged at 3000× *g* for 15 min to remove cells and cell debris. The obtained EVs-containing medium was then mixed with ExoQuick-TCTM Exosome Precipitation Solution (SBI System Biosciences, Palo Alto, CA, USA) in a ratio of 5 to 1 and incubated overnight at 4 °C. The following day, the medium was centrifuged at 1500× *g* for 30 min. The supernatant was carefully aspirated and a pellet was centrifuged for 5 min to remove the remaining liquid. The final pellet containing the precipitated EVs was resuspended in 100 μL or 500 μL (depending on further use) of sterile phosphate buffered saline (DPBS) (ThermoFisher Scientific, Rockford, IL, USA). 

### 4.3. Quantitative and Qualitative Extracellular Vesicles Analysis

The number and size of isolated microvesicles were analyzed using FACSCanto^™^ II cytometer (BD Biosciences, San Jose, CA, USA) and FACSDiva^™^ software (BD Biosciences, San Jose, CA, USA) and TruCOUNT^™^ beads (BD Biosciences, San Jose, CA, USA). Cytometer Setup Tracking (CST) beads and a CST Module were used to calibrate the cytometer. The number of EVs was calculated according to the formula:

$$\frac{\frac{\mathrm{Number}\;\mathrm{of}\;\mathrm{EV}\;\left(\mathrm{annexin}+\right)}{\mathrm{Number}\;\mathrm{of}\;\mathrm{TruCOUNT}\;\mathrm{beads}/\mathrm{gate}}}{\mathrm{events}}\times \frac{\mathrm{Number}\;\mathrm{of}\;\mathrm{TruCOUNT}\;\mathrm{beads}/\mathrm{tube}}{\mathrm{Suspension}\;\mathrm{volume}}$$

The samples of isolated EVs (50 µL) were incubated with 5 µL annexin-V-FITC and 32 µL annexin binding buffer 30 min in the dark. Then 250 µL of annexin binding buffer was added. To calculate EV, TruCOUNT^™^ beads (50 μL) were added immediately before analysis [68,69,70]. Samples without annexin staining were used as the controls to detect non-specific binding. Control samples were incubated in the binding buffer under the same conditions as the stained samples.

### 4.4. RNA Isolation and First-Strand cDNA Synthesis

Total RNA was extracted from thyroid cell lines tested using Purification Kit (EURx, Gdansk, Poland) according to the manufacturer’s protocol. Total RNA concentration and purity was evaluated by measuring absorbance at 260 nm and 280 nm with Synergy 2 Multi-Mode Reader (BioTek, Winooski, VT, USA). Then, 500 ng of RNA was transcribed to cDNA using PrimeScript^™^ RT Reagent Kit (TaKaRa, Kusatsu, Japan) with Oligo dT Primer and Random 6 mers. Reverse transcription reaction was performed on T100^™^ Thermal Cycler (Bio-Rad, Hercules, CA, USA), according to the manufacturer’s protocol. 

Then, gene expression was analyzed using qRT-PCR with Maxima SYBR Green/Fluorescein qPCR Master Mix (Thermo Fisher Scientific, Rockford, IL, USA), 5 nM specific oligonucleotide primers (Genomed, Warsaw, Poland) and 5-fold diluted cDNA samples. Amplification and data analysis were performed using CFX96 Detection System (Bio-Rad) under the following conditions: 95 °C for 30 s; 95 °C for 5 s (40 cycles); 58 °C for 15 s and 72 °C for 10 s. Relative gene expression levels were calculated using β-ACTIN as an internal control for all cell lines except the NTHY cells cultured with isolated EVs, for which 18S rRNA was used. The genes and primers used in this study are listed in Table 1. Each sample was tested in triplicate.

### 4.5. Cell Culture with EVs

NTHY cells (1.7 × 10^5^–3 × 10^5^) seeded in T25 flasks were incubated in medium enriched with 100 µL EVs isolated from thyroid cancer cell cultures suspended in PBS for 48 h. Then, a second portion (100 µL) of EVs isolated from thyroid cancer cells was then added to the culture. After three more days, the cells were used for functional experiments. NTHY cells cultured without EVs were used as a control. The results of all functional experiments were expressed relative to the results obtained for these control cells.

### 4.6. Protein Extraction and Western Blot

Protein isolation and western blotting were performed as previously described [71]. Briefly, crude cell lysates (80–90% confluence) were suspended in RIPA lysis buffer (Thermo Fisher Scientific, Rockford, IL, USA) and total proteins were extracted. Protein concentration was subsequently determined using the BCA Protein Assay Kit (Pierce, Thermo Fisher Scientific, Rockford, IL, USA), and protein lysates were aliquoted and stored at −80 °C until use. 

Then, 20 μg of protein extracts were separated by electrophoresis in a 10% SDS-polyacrylamide gel under reducing conditions and transferred onto a nitrocellulose membrane or PFDV (BioRad). After blocking the non-specific sites for one hour (5% no-fat milk in TBST—0.1% Tween^®^), the proteins were probed with specific primary antibodies overnight at 4 °C (Table 2). The membranes were then washed in TBST and incubated with appropriate HRP-conjugated secondary antibodies (Table 2). Immunoreactive bands were detected using the SuperSignal^™^ West Dura Extended Duration Substrate kit (Thermo Fisher Scientific, Rockford, IL, USA) on Carestream membranes.

### 4.7. Immunocytochemistry (ICC/IF) and Confocal Microscopy Imagine

Cells were cultured for 48 h on glass coverslips, then washed with PBS, fixed with 4% paraformaldehyde for 15 min at room temperature, permeabilized with 0.25% TritonX-100 (10 min), and non-specific sites were blocked with 2% BSA in TBST for 1 h. Slides were then incubated with the specific primary antibody suspended in 2% BSA in TBST (Table 2) overnight at 4 °C. After washing several times with TBST, cells were incubated with the corresponding secondary antibody labeled with Alexa Fluor 594 diluted in 2% BSA in TBST for 1 hour in the dark. To visualize actin filaments, cells were incubated with Phalloidin-FITC (Sigma-Aldrich, St. Louis, MO, USA) and DAPI (4′,6-diamidino-2-phenylindole) for nuclei staining. Finally, cells were observed in a laser scanning confocal microscope LSM 800, AxioObserver Z.1 (Zeiss, Oberkochen, Germany) using ZEN 2.6 software (Zeiss).

### 4.8. Wound Healing Assay

For the wound healing assay, cells (1.5 × 10^5^ cells per well) were seeded into 12-well plates and incubated at 37 °C until a monolayer was obtained. Then, the monolayer was scratched with a sterilized 1–200 μL pipette tip. Cell migration was monitored and three images were taken at different locations in each wound at 0, 12, and 24 h from scratching, using an Observer.D1 fluorescence microscope (Zeiss) and Axio Vison LE software (Zeiss). Migration was assessed by measuring the distance between scratch edges at six different points using ImageJ software (NIH, Bethesda, MD, USA).

### 4.9. Migration and Invasion Assay

For migration assay, chambers with 8-μm pores (Falcon, Corning Live Sciences, NY, USA) were used. The invasion assay was performed in Corning^®^ BioCoat^™^ Matrigel^®^ Invasion Chambers with 8.0-μm pores (Corning Life Sciences, NY, USA). Cells (2 × 10^4^/well), suspended in FBS-free medium, were seeded into chambers. After 24 h of incubation at 37 °C, the chambers were removed from the plate, cleaned and the cells were stained with Diff-Quik^™^ (Medion Diagnostics, Miami, FL, USA). Nine images at different sections of each membrane were taken using light microscope Olympus BX41 (Olympus, Carlsbad, CA, USA). Cells were counted using ImageJ software (NIH, Bethesda, MD, USA).

### 4.10. Proliferation Assay

BrdU Cell Proliferation kit (Merc Millipore, Burlington, MA, USA) was used for the 5 bromo-2-deoxy-uridine (BrdU) incorporation assay, according to the manufacturer’s instructions. Briefly, cells (1200 cells/well) were labeled with BrdU for 5 h, then incubated 30 min with the fixing solution followed by a mouse anti-BrdU antibody for 1 h. After a 30-min incubation with an HRP-conjugated goat anti-mouse antibody, cells were stained with the peroxidase substrate. Afterwards, the absorbance was measured at 450 nm and 550 nm on a Labsystems Multiskan RC micro-plate reader (Labsystems, Helsinki, Finland). 

### 4.11. Cell Viability Assay

Cell viability was analyzed using the CellTiter 96^®^ AQueous One Solution Cell Proliferation Assay (Promega, Madison, WI, USA), according to the manufacturer’s protocol. The assay is based on a tetrazolium compound (3-(4,5-dimethylthiazol-2-yl)-5-(3-carboxymethoxyphenyl)-2-(4-sulfophenyl)-2H-tetrazolium, inner salt; MTS). The reagent was added to cells seeded in 96-well plate at about 1200 cells per well and incubated at 37 °C for 3 h. The quantity of formazan product was assessed by measuring absorbance at 492 nm on a Labsystems Multiskan RC plate reader (Labsystems).

### 4.12. Tube Formation Assay and Analyzis 

A tube formation assay was performed to assess the effect of cancer EVs on human umbilical vein endothelial cells (HUVEC) angiogenesis. The ECM Gel (Matrigel^®^) (Sigma-Aldrich) was added to pre-cooled 96 well-plate and incubated for two hours at 37 °C for polymerization. Subsequently, HUVECs cells (PromoCell, Heidelberg, Germany) were seeded at 3.5 × 10^4^ per well, together with EVs isolated from thyroid cell lines. After a five-hour incubation at 37 °C, the tube formation was photographed under inverted microscope AxioObserver D1 (Zeiss). Tubes’ length and organization were analyzed using Angiogenesis Analyzer (plugin of Image J) (NIH). 

### 4.13. Monocyte Isolation

Monocytes were isolated from the blood of healthy adult human donors using the Bøuym method as described in [72]. Blood samples were processed within two hours. The viability of isolated monocytes was measured by the cell morphology and flow cytometric annexin V-FITC and PI-A apoptosis detection kit II (BD Biosciences, Franklin Lakes, NJ, USA), according to the manufacturer’s protocol. Monocytes were suspended in the RPMI-1640 culture medium that had been supplemented with the 1% antibiotic PSN (penicillin—streptomycin—neomycin) solution (GE Healthcare, Little Chalfont, UK) and used for further research. The study was approved by the local Bioethics Committee at the Centre of Postgraduate Medical Education (no. 26/PB/2018). 

### 4.14. Flow Cytometry Analysis of Extracellular Vesicle Phagocytosis

Monocytes were stained with the rat anti-CD14 phycoerythrin (PE)-labeled anti-body (MøP9; BD Biosciences, San Jose, CA, USA; diluted 1:10). Extracellular vesicles were incubated with the mouse anti–Caveolin-1antibody (Cell Signaling; 1:600) and with the IgG secondary Ab—FITC (Sigma-Aldrich, St Louis, MO, USA; 1:500). The monocyte and vesicle staining procedure was carried out at room temperature for 30 min. Then, the stained monocytes and vesicles were incubated for one hour at 37 °C 5% CO_2_. After washing, the cells and extracellular vesicles were suspended in 300 µL of the PBS buffer and analyzed using the FACSCanto^™^ II and FACSDiva^™^ Software (BD Biosciences, San Jose, CA, USA). A total of 100,000 events were acquired from each sample. The number of monocytes with internalized vesicles was calculated after eliminating the adhered vesicles (samples incubated at 4 °C) according to the following formula: F = a − b.

F: monocytes with internalized EV (%); a: monocytes with internalized and adhered EV (%); b: monocytes with adhered EV [72]. 

### 4.15. Confocal Microscopy of Extracellular Vesicles Phagocytosis

In order to observe phagocytosis under a confocal microscope, human monocyte suspension was adhered onto sterile coverslips placed in 24-well plates. Cells were incubated with EVs for one hour at 37 °C. Then, the coverslips were washed twice with the RPMI culture medium to eliminate non-adherent monocytes. The staining procedure was similar to that used for immunocytochemistry except that 1% PFA was used for fixation and the cells were incubated with the anti-Caveolin1 antibody only for one hour. After that, the slides were mounted with the Fluoromont G^™^ (Sigma-Aldrich, St Louis, MO, USA) cytomatic fluorescent mounting medium. Fluorescence-stained monocytes and EVs were then analyzed using scanning confocal microscope LSM 800, Axio-Observer Z.1 (Zeiss). The Z-stack imaging in ZEN 2,6 software (Zeiss) was used to generate unique 3D images.

### 4.16. Statistical Analysis

Statistical analysis was performed from at least three independent experiments by using the ANOVA test and correlation matrix analysis (GraphPad, Prism 6.00 for Windows, GraphPad Software, San Diego, CA, USA), and *p* < 0.05 was considered as statistical significance.

## 5. Conclusions

In conclusion, we showed that non-fractionated EVs derived from thyroid cancer cells do not affect proliferation, viability, migration and invasion of normal thyroid cells. However, they change the expression of tetraspanins. In contrast to the previously reported stimulatory effect of exosomes, we demonstrated that the total pool of thyroid cancer-derived EVs suppressed angiogenesis. Our results also suggest that the 6-μm EV fraction may exert specific effects on normal thyroid cells as well as on endothelial cells. Further experiments are required to confirm or reject this hypothesis. 

## Figures and Tables

**Table 1:** List of primers used for qRT-PCR.

Gene Name	Nucleotide Sequences
*18S rRNA*	F: 5′ CCAGTAAGTGCGGGTCATAAG 3′ R: 5′ CCATCCAATCGGTAGTAGCG 3′
*β-actin*	F: 5′ GCCGAGGACTTTGATTGC 3′ R: 5′ CTGTGTGGACTTGGGAGAG 3′
*CAV-1*	F: 5′ GCCCTCTTTGAAATCAGC 3′ R: 5′ CAAGTATTCAATCCTGGCTC 3′
*CD9*	F: 5′ GATTGCTGTCCTTGCCATTGG 3′ R: 5′ CTCATCCTTGTGGGAATATCC 3′
*CD63*	F: 5′ CCCTTGGAATTGCTTTTGTCG 3′ R: 5′ CGTAGCCACTTCTGATACTCTTC 3′
*CD81*	F: 5′ TCATCCTGTTTGCCTGTGAG 3′ R: 5′ AGTCAAGCGTCTCGTGGAAG 3′
*CD82*	F: 5′ TGGTGAAACCCCGTCTCTAC 3′ R: 5′ GCCTTATCTAACGCCCTTCC 3′
*CD151*	F: 5′ ATCATCGCTGGTATCCTCG 3′ R: 5′ GTCTCGCTGCCCACAAAG 3′
*Ezrin*	F: 5′ TCTTCGCTGCTGCTGGATAG 3′ R: 5′ GGTGGTAACTCGGACATTGATTG 3′
*Moesin*	F: 5′ TGAGGCTGTGGAGTGGCAGC 3′ R: 5′ CTAGAGGCTGGGTGCCCATT 3′
*Radixin*	F: 5′ GGCAACACAAAGCTTTTGCA 3′ R: 5′ ATATATGCAAAATAACAGCTC 3′
*Alix*	F: 5′ CTGGAAGGATGCTTTCGATAAAGG 3′ R: 5′ AGGCTGCACAATTGAACAACAC 3′

**Table 2:** List of antibodies used for WB and ICC/IF.

Antibodies	WB Dilution	ICC Dilution
Primary antibodies		
Caveolin-1 antibody (D46G3) (rabbit monoclonal, No. 3267, Cell Signaling, Danvers, MA, USA)	1:2000	1:1000
Anti-CD9 (EPR 2949) (rabbit monoclonal, No. ab92726, Abcam, Cambridge, UK)	1:5000	-
Anti-CD9 (P1/33/2) (mouse monoclonal, No. sc-20048, Santa Cruz Biotechnology, Dallas, TX, USA)	-	1:300
Anti-CD63 (MEM-259) (mouse monoclonal, No. MA1-19281, Invitrogen, Carlsbad, CA, USA)	-	1:500
Anti-CD81 (1,3,3,22) (mouse monoclonal, No. sc-7637, Santa Cruz Biotechnology)	1:300	1:400
Anti-CD82 (TS82b) (mouse monoclonal, No. ab59509, Abcam)	1:1000	1:300
Anti-CD151 (H-8) (mouse monoclonal, No. sc-271216, Santa Cruz Biotechnology)	1:500	1:300
Anti-β-actin (AC-74) (mouse monoclonal, No. A2228, Sigma-Aldrich)	1:4000	-
Anti-Ezrin/Radixin/Moesin (rabbit polyclonal, No. ab118572, Abcam)	1:750	1:200
Anti-Alix (3A9) (mouse monoclonal, No. sc-53538, Santa Cruz Biotechnology)	1:500	1:200
Secondary antibodies		
Anti-rabbit HRP-conjugated IgG (No. P0448, DAKO, Glostrup, Denmark)	1:10,000	-
Anti-mouse HRP-conjugated IgG (No. 115-035-146, Jackson ImmunoResearch, Cambridge, UK)	1:10,000	-
Anti-rabbit IgG (Alexa Fluor 594) (No. 8889, Cell Signaling)	-	1:700
Goat anti-mouse IgG (Alexa Fluor 594) (No. ab150116, Abcam)	-	1:900

## Data Availability

The data presented in this study are available in main text and Supplementary Materials.

## Supplementary Materials

The following are available online at https://www.mdpi.com/article/10.3390/ijms23063262/s1. References [73,74,75,76,77,78,79,80,81,82,83,84,85,86,87,88,89,90,91,92,93,94] are cited in the supplementary materials.
