# Supplementary material for: Extracellular Vesicles as Signal Carriers in Malignant Thyroid Tumors?
Source: Int J Mol Sci. 2022 Mar 17;23(6):3262. doi: 10.3390/ijms23063262 (PMC8955189; doi:10.3390/ijms23063262)
Supplement: Supplementary file 1 [file ijms-23-03262-s001.zip › ijms-1600129-supplementary.pdf]

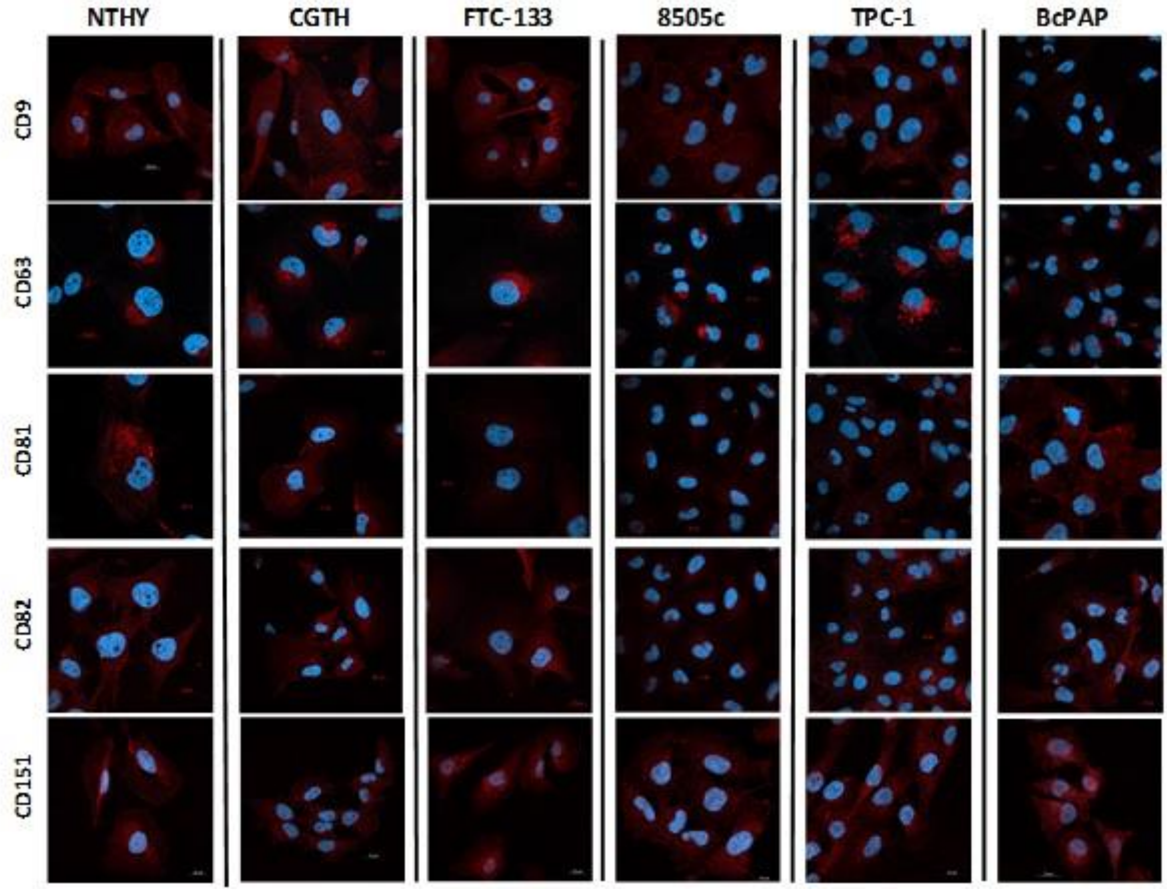

Figure S1. Expression of tetraspanins (CD9, CD63, CD81, CD82, CD151) in different thyroid cell lines observed under confocal microscope [tetraspanins (red), nucleus (blue)] .

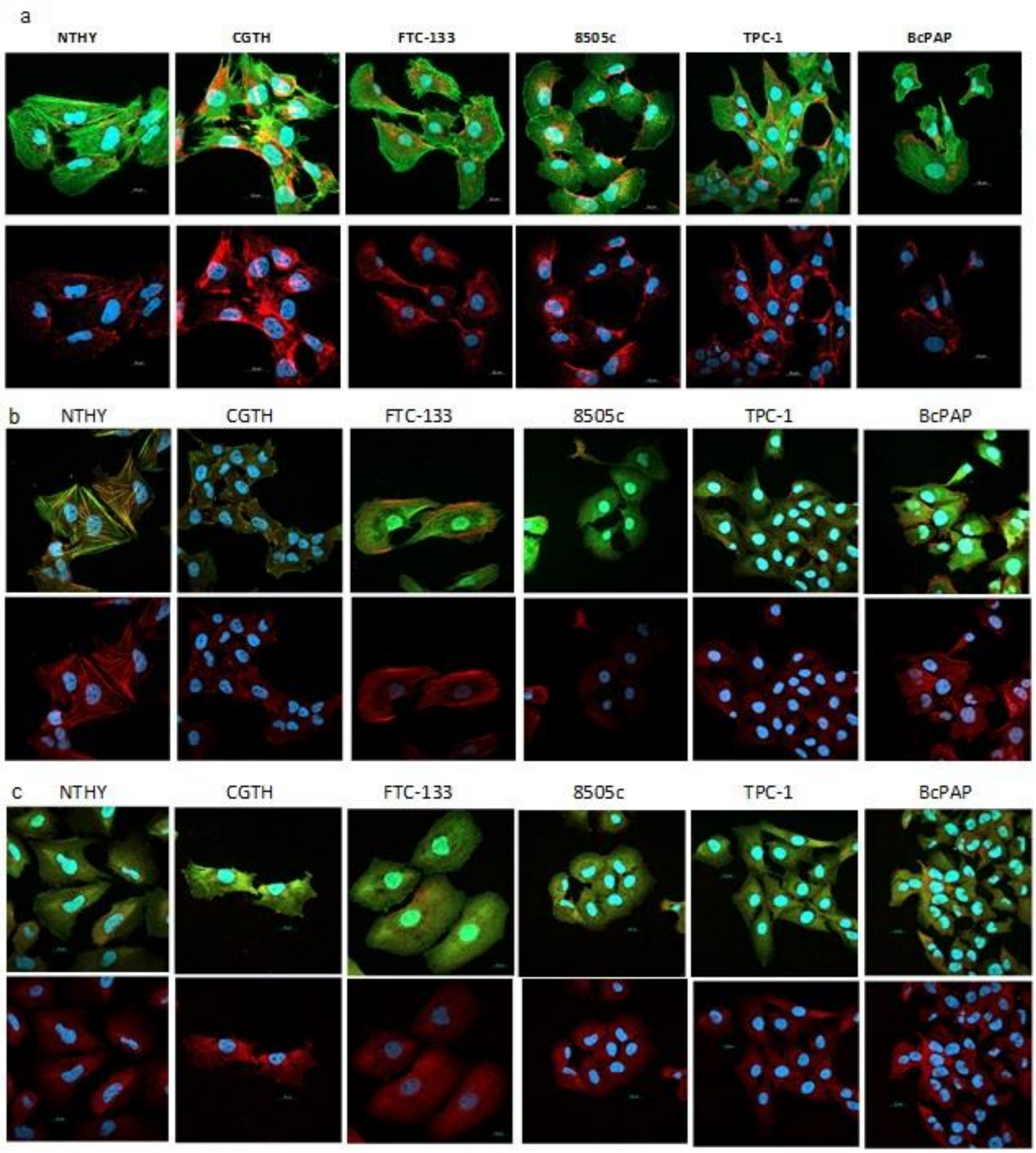

Figure S2. Expression of Caveolin-1 (a), ERM (b), Alix (c) in different thyroid cell lines observed under confocal microscope [protein (red), phalloidin (green), nucleus (blue)].

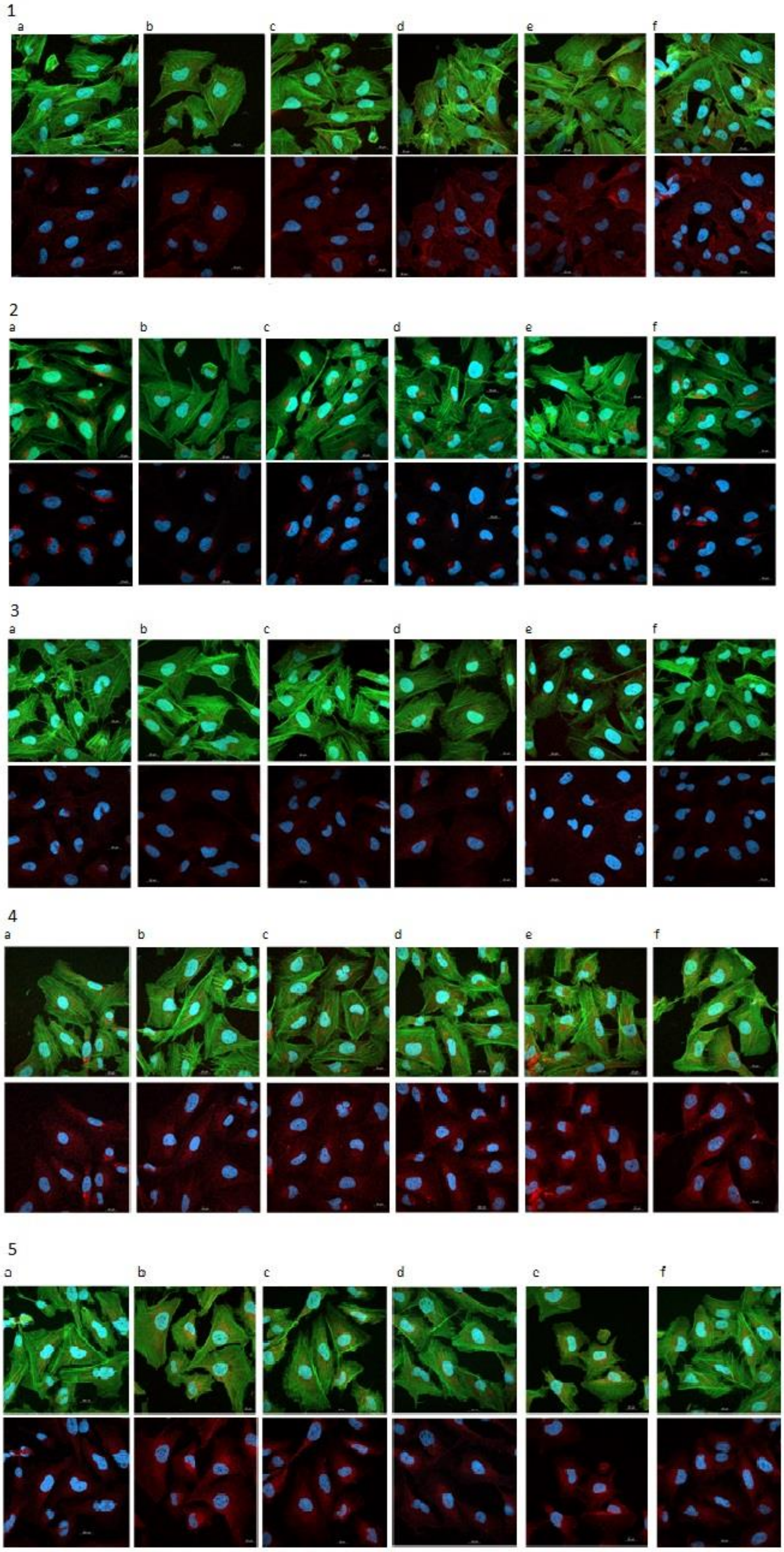

Figure S3. Expression level of tetraspanin protein of CD9 (1), CD63 (2), CD81 (3), CD82 (4), CD151 (5) in NTHY cell line (a), NTHY treated with CGTH (b), NTHY treated with FTC-133 (c), NTHY treated with 8505c (d), NTHY treated with TPC-1 (e), NTHY treated

with BcPAP (f) observed under confocal microscope [tetraspanin (red), phalloidin (green), nucleus (blue)].

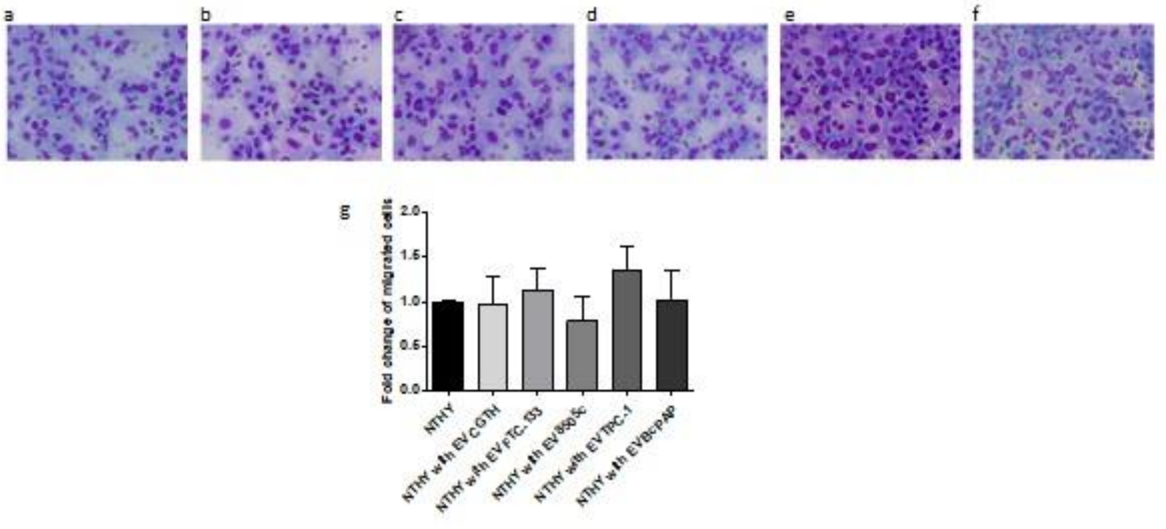

Figure S4. The effect of thyroid cancer EVs on migration of NTHY cells. Upper panel: microscopic analysis of the migrating NTHY. a – NTHY, b – NTHY treated with CGTH EV, c – NTHY treated with FTC-133 EV, d – NTHY treated with 8505c EV, e – NTHY treated with TPC-1 EV, f – NTHY treated with BcPAP EV, Lower panel: g – the percentage of the migrated NTHY cells.

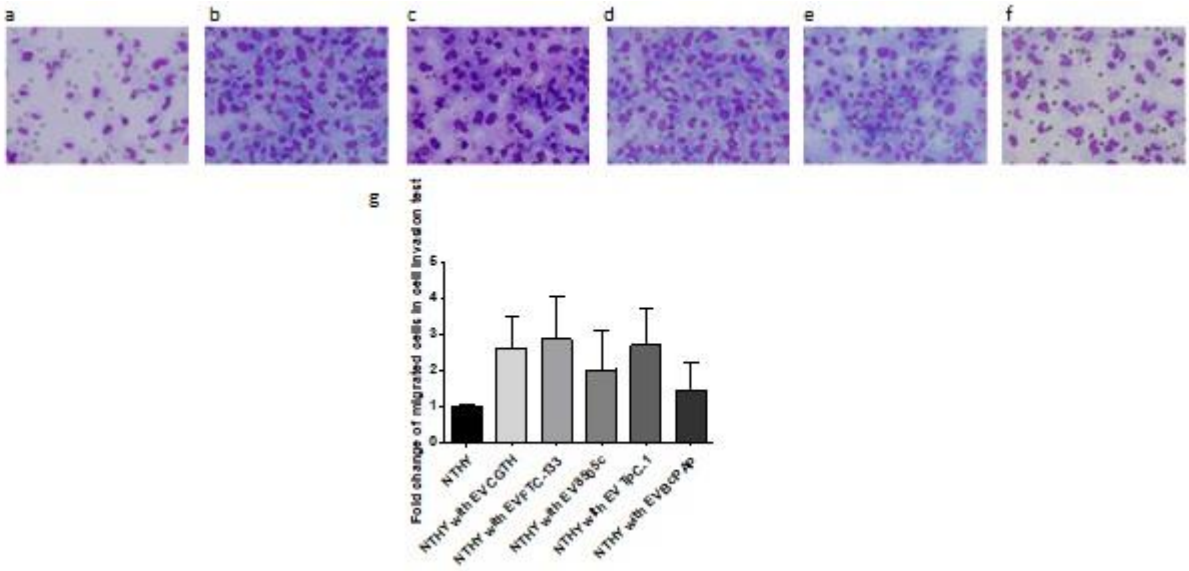

Figure S5. The effect of thyroid cancer EVs on invasion of NTHY cells. Microscopic analysis of the invading NTHY cells: a – NTHY, b – NTHY treated with CGTH EV, c – NTHY treated with FTC-133 EV, d – NTHY treated with 8505c EV, e – NTHY with treated TPC-1 EV, f – NTHY treated with BcPAP EV, Lower panel: g – NTHY cells following invasion.

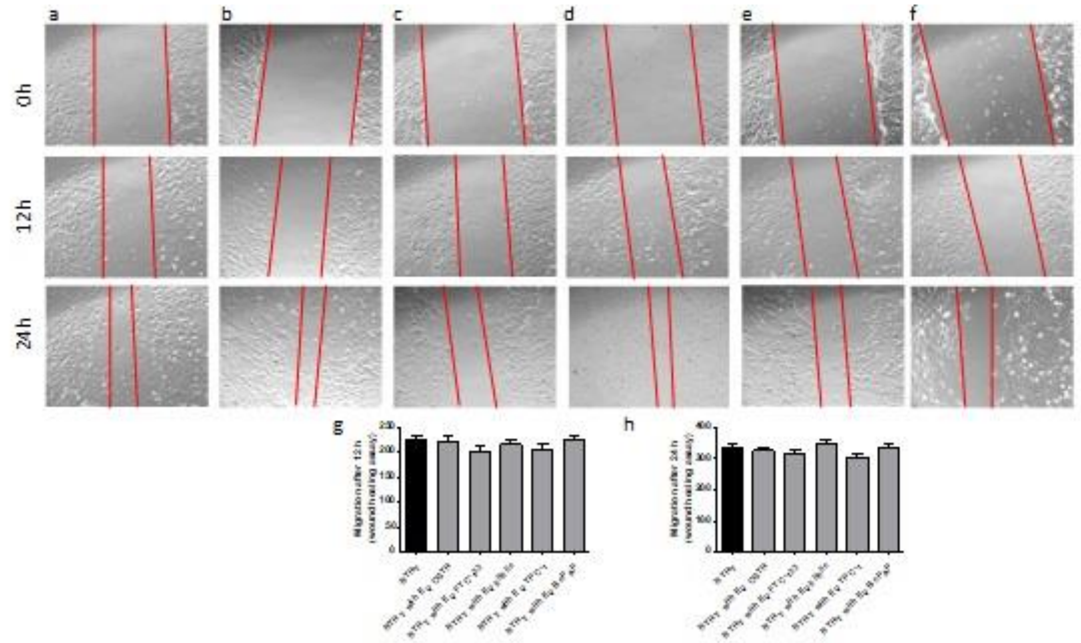

Figure S6. NTHY cells migration in wound healing assay after 12h and 24h: Upper panel: microscopic analysis, a – NTHY, b – NTHY treated with CGTH EV, c – NTHY with FTC-133 EV, d – NTHY with 8505c EV, e – NTHY with TPC-1 EV, f – NTHY with BcPAP. Lower panel: The mean of all distance measurements in μm after 12h (g), 24h (h).

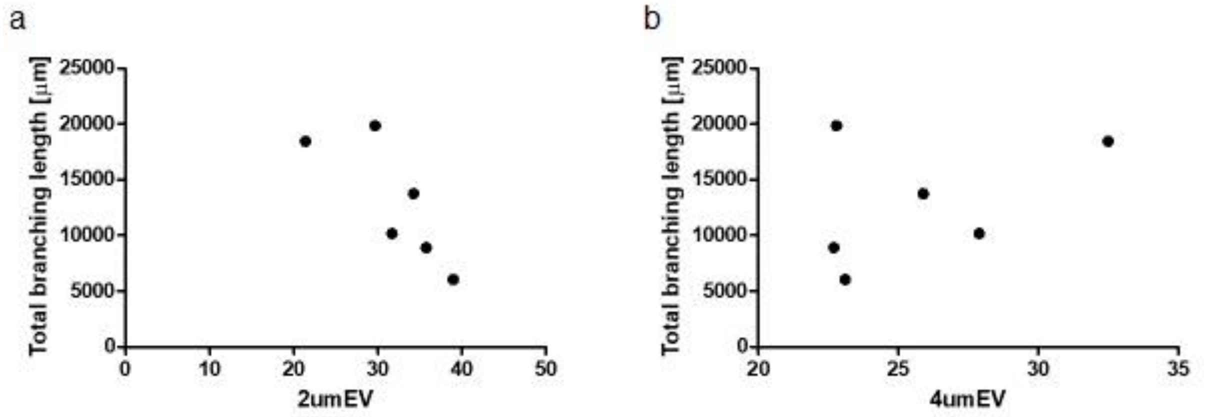

Figure S7. Pearson correlation between the percentage of 2 μm (a) and 4 μm (b) EVs and the pro-angiogenic potential ($p > 0.05$).

Supplementary Table S1. Characteristics of the cell lines used in the study. The information on the mutations has been retrieved from "https://web.expasy.org/cellosaurus" (Accession date: 2022 02 17).

Cell line	Description	References
NTHY	Cell line derived from normal human thyroid follicular cells transformed by SV40 virus	[73–78]
CGTH	Thyroid gland squamous cell carcinoma (previously described as the follicular type), a cell line derived from SW-579; mutations: *TERT* (c.228C>T), *TP53* (p.Ile255Ser)	[71,73,74]
FTC-133	Cell line obtained from a lymph node metastasis of a follicular thyroid carcinoma; mutations: *FLCN* (p.His429Thrfs), *MSH6* (p.Lys1045fs), *NF1* (p.Cys167Ter), *PTEN* (p.Arg130Ter), *TERT* (c.1-124C>T), *TP53* (p.Arg273His),	[74,80–83]
8505c	Cell line derived from anaplastic thyroid carcinoma; mutations: *BRAF* (p.Val600Glu), *NF2* (p.Glu129Ter), *TERT (*c.1-146C>T), *TP53* (p.Arg248Gly)	[83–86]
TPC-1	Cell line derived from thyroid gland papillary carcinoma; mutations: *CDKN2A* (p.Ala68fs), *STAG2* (p.Gln1089Ter), *TERT (*c.1-124C>T), RET/PTC1 gene rearrangement	[83,87–91]
BcPAP	Cell line derived from thyroid gland papillary carcinoma; mutations: *BRAF* (p.Val600Glu), *TERT* (c.1-124C>T), *TP53* (p.Asp259Tyr)	[83,90,92–95]
